# Predicting spring migration of two European amphibian species with plant phenology using citizen science data

**DOI:** 10.1038/s41598-021-00912-4

**Published:** 2021-11-03

**Authors:** Maria Peer, Daniel Dörler, Johann G. Zaller, Helfried Scheifinger, Silke Schweiger, Gregor Laaha, Gernot Neuwirth, Thomas Hübner, Florian Heigl

**Affiliations:** 1 Institute of Zoology, University of Natural Resources and Life Sciences, 1180 Vienna, Austria; 2 Zentralanstalt für Meteorologie und Geodynamik, 1190 Vienna, Austria; 3 First Zoological Department, Herpetological Collection, Natural History Museum Vienna, 1010 Vienna, Austria; 4 Institute of Statistics, University of Natural Resources and Life Sciences, 1180 Vienna, Austria; 5 Naturschutzbund Österreich, 5020 Salzburg, Austria

**Keywords:** Animal migration, Conservation biology, Phenology

## Abstract

Habitat fragmentation is one of the drivers for amphibian population declines globally. Especially in industrialized countries roads disrupt the seasonal migration of amphibians between hibernation and reproduction sites, often ending in roadkills. Thus, a timely installing of temporary mitigation measures is important for amphibian conservation. We wanted to find out if plant phenology can be a proxy in advance to determine the start of amphibian migration, since both phenomena are triggered by temperature. We analysed data of 3751 amphibian and 7818 plant phenology observations from citizen science projects in Austria between 2000 and 2018. Using robust regression modelling we compared the migration of common toads (*Bufo bufo*) and common frogs (*Rana temporaria*) with the phenology of five tree, one shrub, and one herb species. Results showed close associations between the migration of common frogs and phenological phases of European larch*,* goat willow and apricot*.* Models based on goat willow predict migration of common frog to occur 21 days after flowering, when flowering was observed on 60th day of year; apricot based models predict migration to occur 1 day after flowering, observed on the 75th day of year. Common toads showed weaker associations with plant phenology than common frogs. Our findings suggest that plant phenology can be used to determine the onset of temporary mitigation measures for certain amphibian species to prevent roadkills.

## Introduction

More than 70% of the world’s amphibian populations are in decline and more than two-thirds of amphibian species in the European Union countries have an unfavourable conservation status^1,2^ Modification, loss and fragmentation of habitats are among the most important causes of local declines^3^. In industrialized countries roads cut through landscapes, fragment habitats and disrupt amphibian migration routes in temperate regions resulting in millions of amphibians killed on roads each year^4,5^. In Austria roadkills are recorded in a citizen science project, and are found to be particularly high in the vicinity of amphibians preferred habitats^6,7^. To avoid amphibian roadkills many conservation measures such as protection fences have been adopted^8^. These temporary fences are installed along roads; amphibians migrating from hibernating to the spawning waters fall into buckets inserted along the fences and are released on the other side of the road often by the help of volunteers^9^. Critically for the success of these measures is a timely installation and the daily monitoring of these mitigation measures, in order to minimize roadkill^9–11^. However, daily monitoring is time-consuming^11^ and timely installation is complicated because the timing of amphibian migration can vary considerably from year to year^12^.

Understanding the timing of seasonal migrations in amphibians has received considerable attention^13–16^. Amphibian species vary greatly in their migration behaviour^17^. Bufonidae and Ranidae in temperate regions are so-called “explosive breeders” forming large agglomerations with thousands of specimens during spring migration^18^. As a result, roads that separate overwintering areas from breeding ponds can pose a serious threat to local amphibian populations because they restrict the seasonal migration^8,19^. In studies conducted in European countries spring migration of common toads has been found to correlate with the mean daily temperature 40 days preceding migration^20^ or with average temperatures in March^21^ or April^22^. Similar findings are known for common frog with spawning dates correlating with air temperature in winter and early spring^21^ or the temperature sum in February and March^12^. Besides temperature, also daylength^20^ and rainfall^13,23^ have been linked to spring migration of common frog and common toad in past studies in temperate regions. However, temperature is found to be the main driver of amphibian phenology, while the impact of precipitation is considered weak^24^.

Another phenomenon that is mainly influenced by temperature (and also daylength) is plant phenology including bud break, leaf unfolding or flowering^25–27^. Plant phenology in temperate regions may also be influenced by precipitation, although the extent of such effects is considered to be smaller than that of temperature and daylength^28^.

Hence, the objective of this study was to examine whether phenological phases of plants are associated with amphibian migration patterns. Possible associations would allow to make predictions about the start of local amphibian migrations which is currently mainly determined by personal experience of the people involved. Indications on the start of amphibian migration could be especially helpful for volunteers in nature conservation with limited experience and for regions without observational values yet, in order to timely install temporary amphibian mitigation measures. Further, such an approach would be advantageous for amphibian conservation associations as monitoring plant phenological phases is easy to conduct and data on phenological phases are usually available^2,29^. Another advantage of using phenological phases is that they are considered to reflect the combined effect of temperature and other meteorological variables and daylength and should, therefore, allow better predictions of the onset of amphibian migration than the factor temperature alone.

To investigate this, we analysed 3751 observations of two amphibian species and 7818 observations of flowering and leaf unfolding of seven plant species collected in Austria between 2000 and 2018. Three datasets were collected within citizen science projects (“naturbeobachtung.at” by Naturschutzbund Österreich^30^, “Roadkill” by University of Natural Resources and Life Sciences^31^, “Phenowatch” by Zentralanstalt für Meteorologie und Geodynamik ZAMG^29^); the fourth project included both citizen science and expert data (“Herpetofaunistic Database” by Natural History Museum Vienna^32^). We used robust regression models to test whether we can predict the onset of amphibian migration with plant phenology.

## Results

### Start of amphibian migration and plant phenological phases

We determined the phenological phases of amphibians and plants for the years 2000–2018 in three different climatic regions of Austria referred to as warm, moderate and cool region (Supplementary Tables S1 and S2, Supplementary Fig. S1).

**Figure 1:**
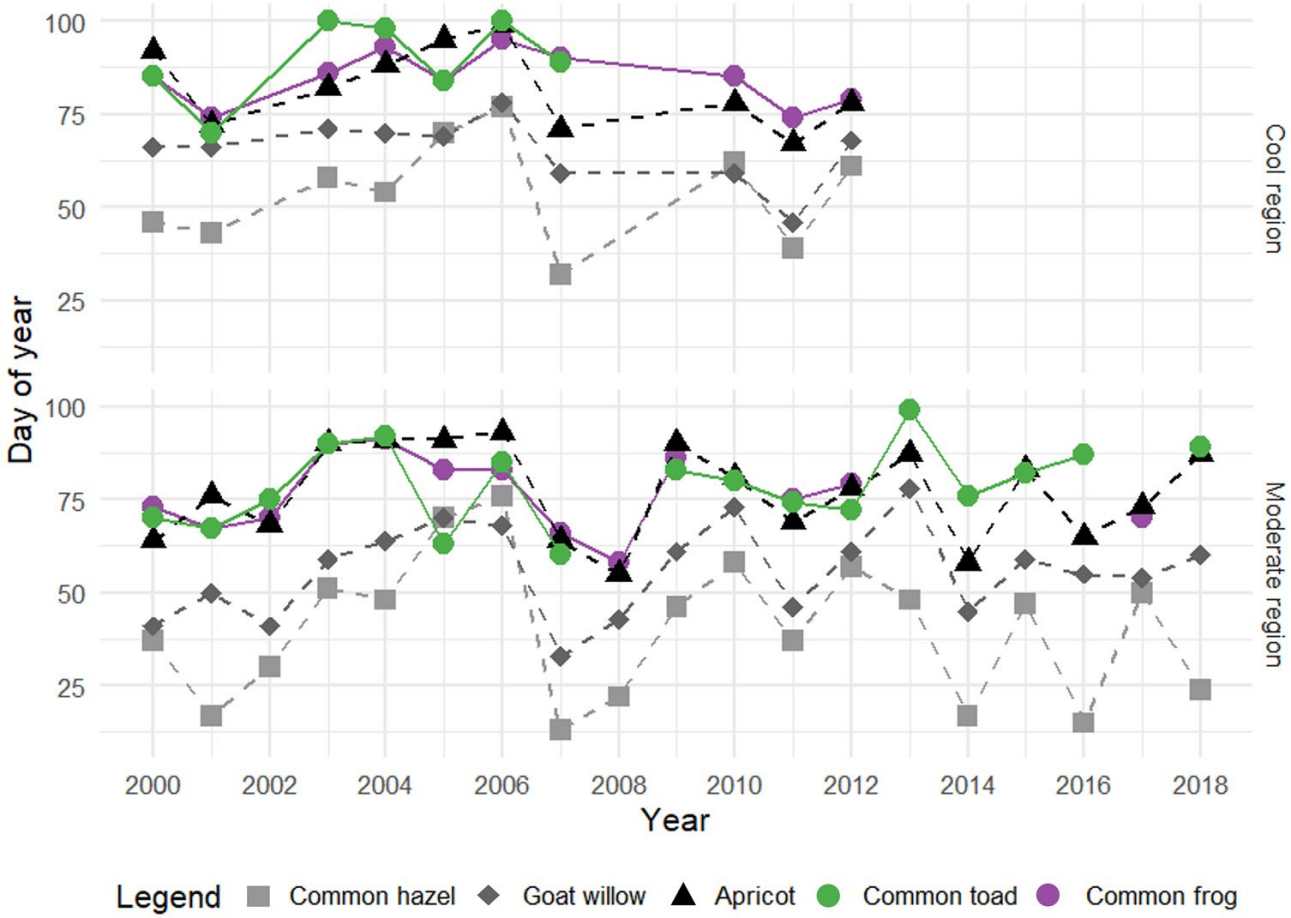
Earliest migration observation of common toads and common frogs and plant phenological phases of common hazel, goat willow and apricot in cool and moderate climate regions in Austria between 2000 and 2018, data missing for some years. Time series of European larch, horse-chestnut, silver birch and snowdrop in Supplementary Fig. S3.

**Figure 2:**
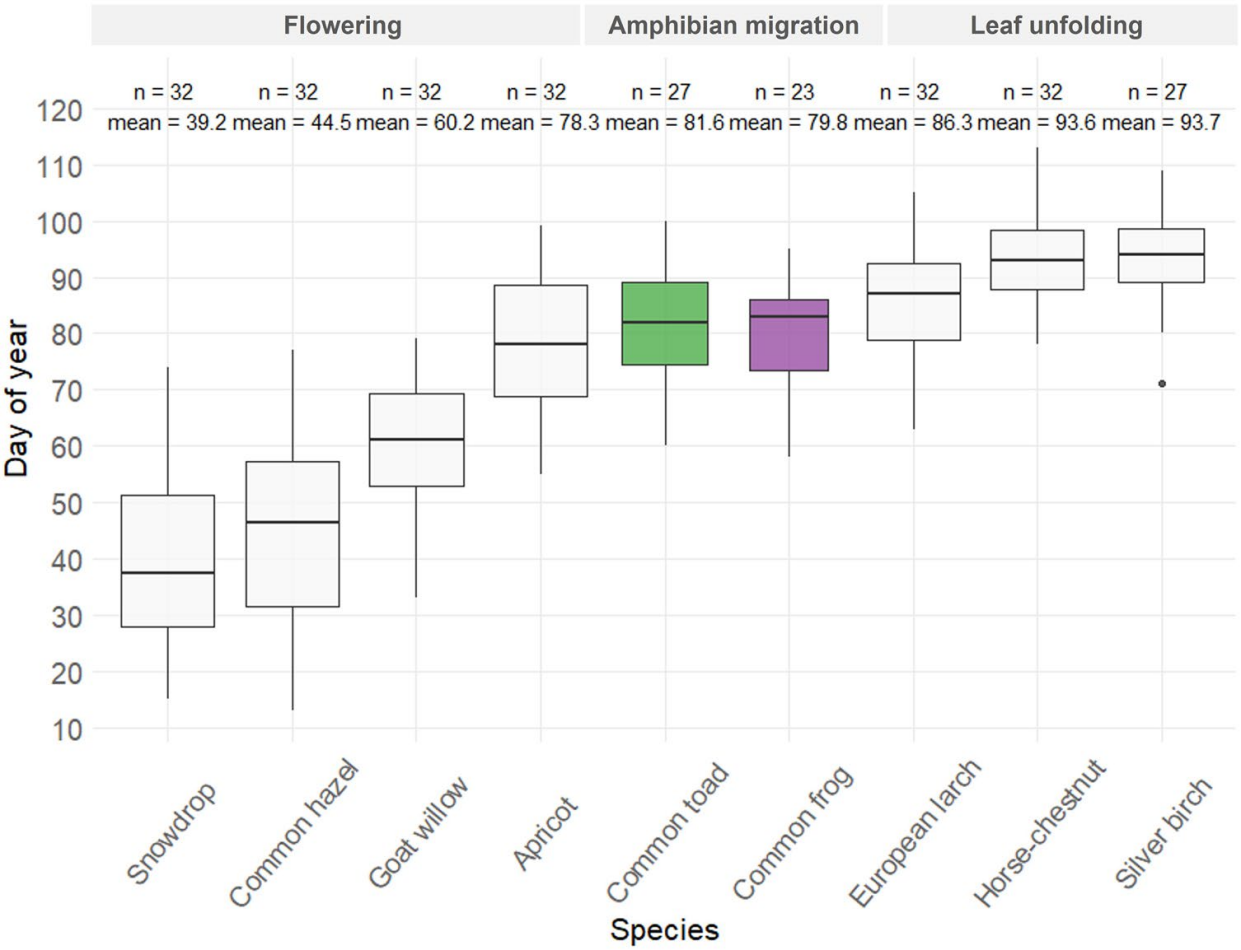
Onset of phenological phases flowering (snowdrop, common hazel, goat willow and apricot) and leaf unfolding (European larch, horse-chestnut, silver birch) and migration start of common toads and common frogs between the years 2000–2018 in Austria.

Migrations of common toads and common frogs were observed across a time span of 37 days; earliest record of common frogs was on February, 27th 2008 (58th day of the year) and the latest record was on April, 5th 2006 (95th day of the year; Fig. 1). Earliest documented migration of common toads was on March, 1st 2007 (60th day of the year) in the moderate region. The latest recorded start of migration of common toads was in 2003 and 2006 on April, 10th (100th day of the year), both in the cool region. Migration of common frogs started only about 2 days earlier (Mean = 80th day of the year) than migration of common toads (Mean = 82nd day of the year) on average (Fig. 2). Common frog and common toad migration started significantly earlier in the moderate region (median = 76th day of the year) than in the cool region (median = 85th day of the year) (*p* < 0.05)) (Supplementary Fig. S2). Due to the limited data available, it is not possible to compare the warm region (Median = 78th day of the year, n = 3) with the other regions.

Regarding plant phenology, flowering of snowdrop and common hazel flowered first in January or February, at the beginning of March at the latest—about 40 days before the start of the first amphibian migrations (Fig. 2). Goat willow started flowering in February at the earliest—about 20 days before amphibian migrations. Albeit not significantly different, flowering of apricot appeared slightly earlier than migration of common toads and common frogs. European larch, horse-chestnut and silver birch unfolded their leaves around the end of March to the beginning of April, thus happening later on average than amphibian migration. In general, leaf unfolding phases showed a lower temporal variance compared to flowering phases.

No significant shift of the starting dates for amphibian migration and plant phenology phases was found across the study period (Mann–Kendall test, *p* > 0.05, Fig. 1). Both, amphibian migration and plant phenological phases showed similar temporal patterns for cool and moderate regions, although with staggered starting dates.

### Associations of amphibian migration with plant phenology

**Figure 3:**
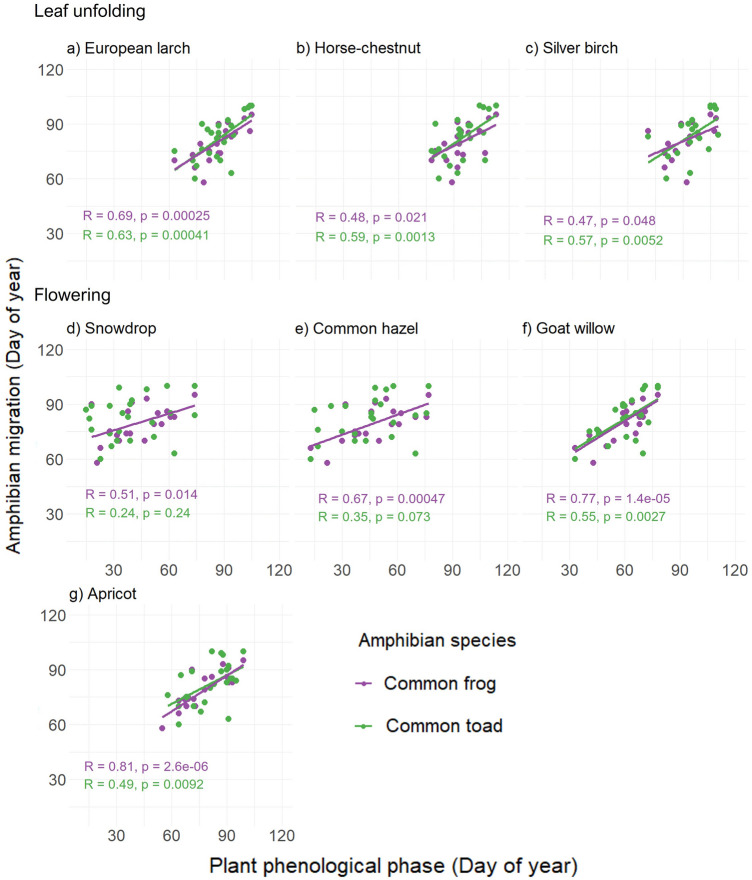
Correlations between the start of the migration of common toads and common frogs and onset of leaf unfolding of European larch (**a**), horse-chestnut (**b**), silver birch (**c**) and start of flowering of snowdrop (**d**), common hazel (**e**), goat willow (**f**) and apricot (**g**).

Start of the seasonal migrations of common toads and common frogs was significantly correlated with five plant phenological phases analysed (*p* < 0.05; Fig. 3). Exceptions were the correlations between the start of migration of common toads and the flowering of snowdrop and between the start of migration of common toads and the flowering of common hazel.

### Predicting the start of amphibian migration

We considered plants with phenological phases starting before amphibian migration and sufficient correlation to amphibian migration as suitable for predicting amphibian migration.

**Figure 4:**
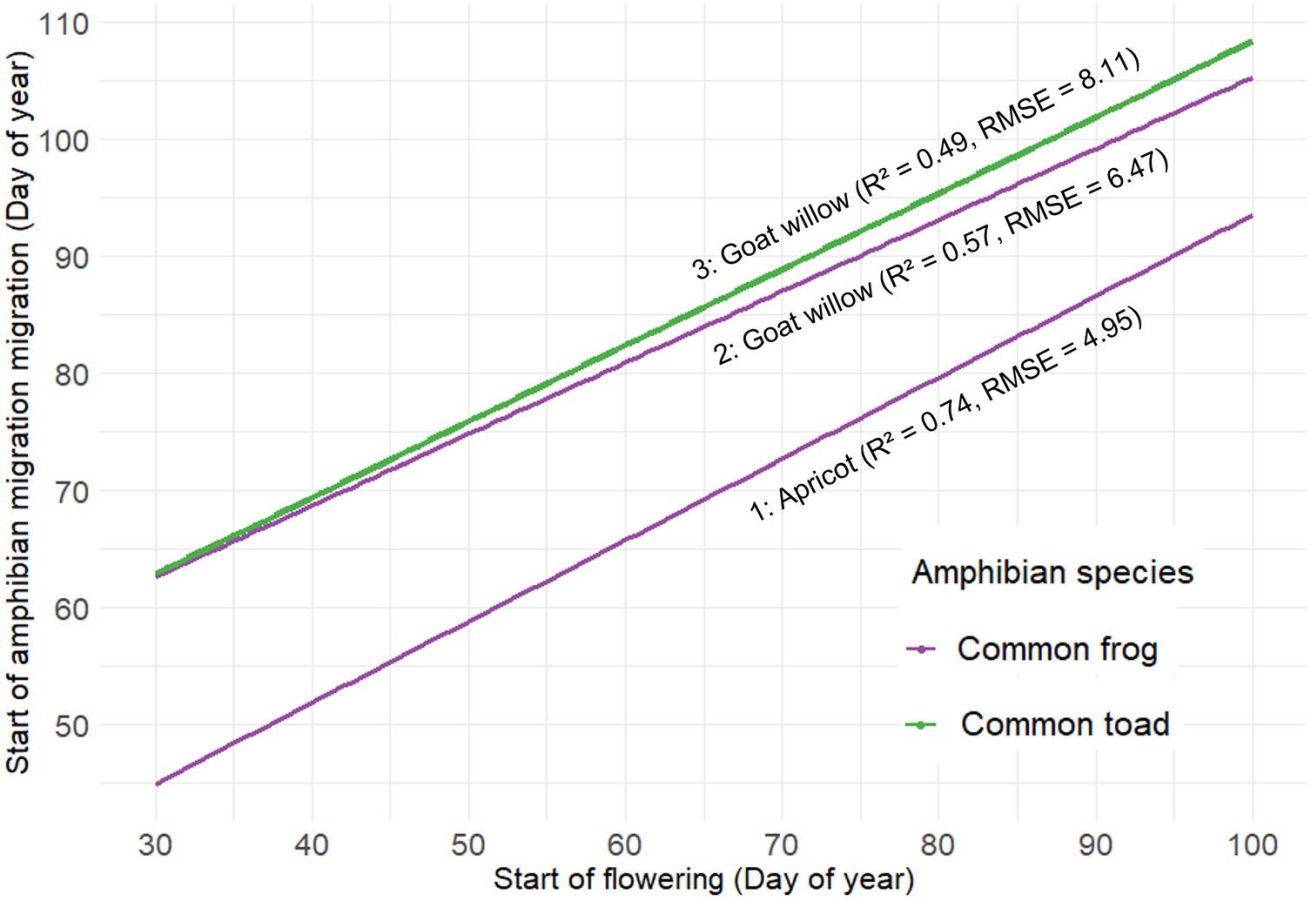
Regression models to predict migration of common frog (model 1 and 2) and common toad (model 3) with flowering of plants labelled. More specific predictions for moderate and cold regions can be derived from models in Supplementary Table S3.

Three models were best in predicting the migration of common frogs or common toads (Fig. 4). Especially well adapted to the data is model 1 with apricot; apricot and goat willow showed significant regression coefficients (*p* < 0.05) for the migration of common frogs and confirmed the results of the correlation tests. For the prediction of the migration of common toad, goat willow showed the best results (model 3). Adding other plants as predictors to existing models based on goat willow and apricot did not significantly improve the model. Additional, weaker model outputs for common frogs and common toads and a null model to which we compared our models can be found in Supplementary Table S3.

## Discussion

Our analysis of large datasets of amphibian observations and plant phenology over a time span of 18 years showed that the start of amphibian migrations can be predicted with plant phenology data. Using robust regression modelling we were also able to predict the interval between plant phenology events and the likely onset of amphibian migration. This can be especially practical in timing the installation of temporary mitigation measures to avoid amphibian roadkills as plant phenology is more easily assessed than the start of amphibian migration and plant phenology data are often reported by weather agencies.

### Association of amphibian and plant phenology

We found great similarities in the phenological phases of the selected plant and amphibian species: five out of the seven selected plant species showed a high and significant correlation either with the migration of common frogs or common toads or both amphibian species. Migration of common frogs and flowering of apricots occured almost simultaneously. Also, phenological phases of goat willow, common hazel and European larch showed a high correlation with migration of common frogs or common toads. These correlations suggest common relationships of the studied phenological phases on meteorological factors. Others have shown for both the examined amphibian species and plant species, that mainly temperature influences the timing of phenological activity: amphibian migration has been shown to be influenced by temperature in winter^21^, alternatively by temperatures in February and March^12,21,33^ or April^22^ or by temperatures, 40 days preceding migration^20^. Likewise, plant phenological phases in spring are found to be especially influenced by daily mean temperatures of the preceding months in temperate latitudes^34,35^. Additionally day length is assumed to control the seasonal cycle of plants besides temperature^26,27^ and is also considered to prevent breeding activity of common toads from occurring too early in the year^20^. It can therefore be concluded that the observed correlations between amphibian migration and plant phenological phases, could be a result of similar dependencies on temperature and daylength.

The lowest correlation was found between flowering of snowdrop and migration of common toads and between flowering of common hazel and migration of common toads. Flowering of these two plant species was 37–42 days prior to common toad migration suggesting that this quite large time difference could explain the weak correlation. Another explanation for the weak correlation might be the great year to year variability of flowering of snowdrop and common hazel, which was also found for other early spring events and is assumed to be due to greater temperature variability in winter^36^. This is in line with others, who found that correlation strength between phenological events and heat accumulation increases as the season progresses^37^. Surprisingly, flowering of snowdrop and common hazel still show relatively high correlations to migration of common frogs compared to migration of common toads, although common frogs start migrating nearly at the same time as common toads on average. In general, correlations and regression modelling with plant phenology showed better results for common frogs than for common toads indicating that additional species-specific factors affect both plants and amphibians.

### Predicting amphibian migration for nature conservation actions

A better understanding of the timing of amphibian migrations is important for the conservation of amphibian species, as several mitigation strategies, such as the widely used fence-bucket-method depend on the prediction of the onset of amphibian migrations. Our results suggest that amphibian migrations can be predicted without any technical equipment based on easy-to-recognize plant phenological phases. This could help volunteer conservationists to set up protective measures for amphibians in a timely fashion. In the study region Austria, a great advantage of plant phenological observations is that they can be carried out by laypersons as the surveyed plants are widely distributed throughout the country^38^. The flowering phases of the plants used in the models, especially the flowering of apricot, are easily identifiable. Another advantage over predicting amphibian migration with temperature data is the fact that other possible influencing factors, like daylength as shown above, are integrated in plant phenology.

From the results of the models, it can be concluded that apricot and goat willow are most appropriate for predicting the amphibian migration of the common frog. If both plant species are present in a region, apricot is preferred to predict common frog migration because it is more accurate than the model including goat willows. According to the distribution of phenological observations by the Austrian meteorological agency apricot trees are prevalent in most parts of the study area^29^.

Based on the most significant models between plant phenology and amphibian migration we can derive the following relationships. If apricot flowering is observed around the 60th day of year (March 1st), common frog migration is likely to begin about 5 days [95% CI: 2–9] later; if flowering is observed around the 70th day of year (March 11th) migration is predicted to begin 3 days [95% CI: 0–5] later; and if flowering is observed around the 80th day of year (March 21st), migration is predicted to begin at about the same time as the apricot flowering [95% CI: − 2–2]. The latter relationship is not optimal, as it is more likely that the start of amphibian migration is missed if flowering occurs at the same time or even after amphibian migration. It could therefore be helpful to observe the buds of apricot as the flowering of apricot becomes apparent in the buds already a few days before the actual flowering starts^39^.

In years where the development of plants is on average later, or for regions where apricot is absent, goat willow can help predict common frog migration by considering the following relationships. If goat willow flowering is observed on the 50th day of year (February 19th)/70th day of year (March 11th), common frog migration is predicted to begin 25 [95% CI: 22–28]/17 [95% CI: 14–20] days afterwards. This relationship is somewhat less accurate than the one based on flowering of apricot but may be still useful to install amphibian mitigation measures.

Although we assessed these relationships across a variety of locations, three climatic regions and over 18 years local effects may of course change the modelled relationships. Breeding phenology of common frogs in temperate zones could be influenced by population size and pond temperature^16^, or genetic differences and local adaptation^40^.

## Conclusions

The strong associations between migration of common frogs and plant phenology indicate similar dependences of amphibians and plants on temperature and day length. Predictions for common toads turned out to be less accurate indicating that additional plant species could be assessed. Our findings suggest that reports of plant phenology through weather agencies might be used to support volunteers engaged in roadkill mitigation measures for amphibian conservation.

## Methods

### Study area

Austria is located in the transition zone from oceanic to continental climate^41,42^. The average annual temperature is 7.3 °C (reference period 1987–2016). Austria shows high fragmentation values in non-alpine areas and has a particularly dense road network with a total length of 125,000 km^43–45^. Since 1984 amphibian mitigation measures using the temporary fence-bucket method have been implemented across the country^46–48^ and are in use until today^10,11,49^. At the start of the migration, a fence that is insurmountable for amphibians (about 40 cm high) is set up parallel to the road on the amphibians’ arrival side. Along this fence, buckets are buried about every 20 m at ground level forcing migrating amphibians to fall into the buckets as they walk along the fence. Buckets are then carried to the other side of the road by volunteers and amphibians therin released^9^.

### Data sources

In the context of this study a cooperation with four citizen science projects of four Austrian institutions (Natural History Museum Vienna, Naturschutzbund Österreich, University of Natural Resources and Life Sciences Vienna, Zentralanstalt für Meteorologie und Geodynamik ZAMG) was started, and the data used, that was collected within the framework of the projects (Table 1). The amphibian observation data originate from three different projects. Data on the distribution of amphibian species in Austria has been documented in the Herpetofaunistic Database from the Natural History Museum Vienna since 1982 and since 2002 citizen scientists are contributing amphibian observations. About 80% of the data that we used from the Herpetofaunistic Database were collected by biologists through mapping (e.g., monitoring within the framework of the Habitats Directive, for environmental impact assessments, annual monitoring of amphibian migration routes), the rest are citizen science data, most of them recorded during monitoring of amphibian mitigation measures and all of them checked by biologists. The projects Roadkill and naturbeobachtung.at collect presence-only data observed by citizen scientists via smartphone app or website^30,31^. For our study we selected observations that were verified by experts in the project team. Data for plant phenological phases originates from the phenological monitoring program “PhenoWatch”^29^, in which citizen scientists report the seasonal development of indicator plants. Participants in the project are asked to observe the selected plant species at a selected location with the help of observation guidelines over a period as long as possible and to submit the observed phenological events to PhenoWatch. Data from PhenoWatch contributes to the European Phenological database (pep725.eu)^50^. Data from the four projects have already been included in several individually published studies^6,51–53^.

**Table 1:** Data sources of amphibian observation data and plant phenological phases.

				
	Herpetofaunistic Database	naturbeobachtung.at	Roadkill	PhenoWatch
Carried out by…	Natural History Museum Vienna	naturschutzbund	Institute of Zoology	ZAMG
Data type	Amphibian sightings	Amphibian sightings	Amphibian signings	Plant phenolog. data
Number of observations	2126	1286	339	7818
Time span	2000–2018	2006–2018	2014–2018	2000–2018
Parameters assessed	Survey method Sighting Date Number of individuals Development stage Species Coordinates Habitat Elevation	Species Gender Development stage Dead/Alive Number of individuals Sighting Date Location By App (yes/no) Photo documentation (Not mandatory)	Date and Time Coordinates Photo documentation Species Number of individuals Type of locomotion (and frequency)	Coordinates Elevation Date Species

### Studied amphibian species and their phenology

Migration of the two species common toads *Bufo bufo* L. and common frogs *Rana temporaria* L. were studied. Common toads are the most widespread amphibian species in Austria occurring in all parts of the country^54^. Common frogs are widespread in high mountains, but are missing in the eastern lowlands^55^. Common frogs start to migrate between the end of February and March, and common toads from March to April^55,56^.

### Studied plant species and their phenology

Plant phenological phases leaf unfolding and flowering of five tree species (Horse-chestnut *Aesculus hippocastanum* L., silver birch *Betula pendula* Roth, European larch *Larix decidua* Mill., apricot *Prunus armeniaca* L. and goat willow *Salix caprea* L.), one shrub species (common hazel *Corylus avellana* L.), and one herb (snowdrop *Galanthus nivalis* L.) were studied. Those plants were chosen, as they are part of the phenological observation program of Zentralanstalt für Meteorologie und Geodynamik and are considered to be widely distributed in large parts of Austria^38^. The plants show either the phenological event "flowering" (flowers are open in at least three places of the observed object) or “leaf unfolding” (the first leaves are fully unfolded, unrolled and show their final shape) in spring.

### Defining amphibian migration

Amphibian migration in general is defined as movement, primarily by adults, toward and away from aquatic breeding sites. In contrast to amphibian dispersal, amphibian migrations occur with a higher number of individuals^56^. Additionally longer distance seasonal breeding migrations are non-random and highly directed toward breeding sites, what distinguishes them from juvenile movement^5^. Nevertheless, we are not aware of a definition or limit in literature for the number of individuals above which amphibian migration can be assumed. The boundary limit for this study was defined in the following way:

Every observation that counts > 10 individuals is considered an amphibian migration event. An amphibian observation also counts as a migration, if there are other observations within an area of 1 km^2^ and within 7 days, and if the sum of the individuals reported is > 10.

### Determining the start of amphibian migration

In sum, 3751 records of amphibian observations were used for the analysis and were transferred into the geographic information system (GIS) ArcGIS 10.6.1 (ESRI, Vienna, Austria). To identify amphibian migrations in these records we applied a spatial criterion (Are there amphibian observations within 1 km^2^ of the examined observation?); If true: a temporal criterion (Are there amphibian observations within 7 days of the date of the examined observation?); If true: a quantitative criterion (Is the sum of individuals ≥ 10?). The defined criteria were applied to all amphibian observations and 2116 observations met all criteria and were therefore considered part of an amphibian migration.

As amphibian migration and plant phenological phases are mainly influenced by temperature^24^ we deduced that amphibian migration will start earlier at places with higher average temperatures. We defined regions based on the Austrian digital climate map—mean annual air temperature (MAT) from 1971 to 2000^57^, and therefore ensured that also the start of amphibian migrations and plant phenological phases at average colder places is represented. The “Alpine region” (MAT < 5 °C, ~ 1200–3500 m above sea level) includes Alpine regions without much amphibian migrations and is therefore no further addressed. The “cool region” (5 °C ≤ MAT < 7 °C, ~ 500–1200 m asl) is located mostly in the north of upper and lower Austria, but also contains places in the proximity of the Alps. The “moderate region” (7 °C ≤ MAT < 9 °C, ~ 300–500 m asl) comprises mainly the Alpine foothills and parts of the foothills in the east. The “warm region” (MAT ≥ 9 °C, ~ 100–300 m asl) is mainly found around the Vienna Basin (Supplementary Fig. S1). All amphibian observations that were classified as migrations were assigned to one of the three climatic regions. As our dataset did not contain absence data, we could not assume that the earliest observation of amphibian migration at a site necessarily marked the start of migration at that site. Therefore, the earliest amphibian migration per year and per region was assumed to most closely reflect the true start of the amphibian migration and determined to define the start of amphibian migration in this specific region; provided that there were four or more amphibian migrations found in that year and region. In sum we identified 50 starting times of amphibian migrations—27 for common toad, 23 for common frog.

### Determining the start of plant phenological phases

To determine the start of the plant phenological phases, all reports of the phenological phases were assigned to one of the three climatic regions as well. As for the amphibian observations, only the first phenological event per year was selected, and determined to define the start of the phenological phase (leaf unfolding or flowering). Except for silver birch, which had no recorded observations in the years 2000–2002, we could identify starting dates for all plant species for all years.

### Data analysis

Data was analysed in R studio 3.6.1^58^. In order to determine if the prerequisites for subsequent tests and model analysis are fulfilled, the basic structure of the data was examined, using histograms and Q-Q-plots. A Shapiro–Wilk-Test was conducted to test for normal distribution of amphibian migration and plant phenological data. The chronology of all phenological phases was analysed, both graphically and by comparing mean and median values. Also, paired t-tests were conducted, to compare amphibian migration in the different climatic regions. The non-parametric Mann–Kendall test was conducted to find out if there was a trend to an earlier or later occurrence of the phenological phases. To evaluate the linear association between the start of amphibian migration and the plant phenological phases correlation tests (“Pearson's product-moment correlation”, two-tailed, n_common frog_ = 23, n_common toad_ = 27) were conducted, and the packages “ggplot2” and “ggpubr” were used for graphic presentation. The model type lmrob() from the package “Robustbase” was used to create prediction models. Models were created with one or maximum two plant phenological phases as predictor variables. Mean Annual Temperature MAT was included, to address the effects of specific sites. Leverage points were detected manually with Cook’s distance and scrutinised when the value was greater than 4/N. To rule out multicollinearity the Variance Inflation Factor (VIF) was used. Leave-one-out cross-validation (LOOCV) was used to evaluate the performance of the prediction models and all created models were compared to a null model in terms of Root Mean Square Error RMSE and Adjusted R^2^. All statistical tests were performed by using a significance level of 0.05.

## Supplementary Information

Supplementary Information 1.

## Data Availability

The dataset generated and analysed during the current study (Start of amphibian migration and plant phenological phases in Austria between 2000 and 2018) is available in the Supplementary File.
