# Supplementary material for: Predicting spring migration of two European amphibian species with plant phenology using citizen science data
Source: Sci Rep. 2021 Nov 3;11:21611. doi: 10.1038/s41598-021-00912-4 (PMC8566551; doi:10.1038/s41598-021-00912-4)
Supplement: Supplementary file 1 — Supplementary Information 1. [file 41598_2021_912_MOESM1_ESM.pdf]

Supplements

Predicting spring migration of two European amphibian species with plant phenology using citizen science data

Maria Peer*[1], Daniel Dörler[1], Johann G. Zaller[1], Helfried Scheifinger[2], Silke Schweiger[3], Gregor Laaha[4], Gernot Neuwirth[5], Thomas Hübner[2], Florian Heigl[1]

[1] Institute of Zoology, University of Natural Resources and Life Sciences, 1180 Vienna, Austria
[2] Zentralanstalt für Meteorologie und Geodynamik, 1190 Vienna, Austria
[3] First Zoological Department, Herpetological Collection, Natural History Museum Vienna, 1010 Vienna, Austria
[4] Institute of Statistics, University of Natural Resources and Life Sciences, 1180 Vienna, Austria
[5] naturschutzbund Österreich, 5020 Salzburg, Austria
*Corresponding author: maria.peer@boku.ac.at

Supplementary Table S1. Start of amphibian migration of common toads and common frogs in three climatic regions in the years 2000-2018. No data for common frogs in the warm region. Entries marked in bold indicate amphibians were sighted in several places that day, therefore two or more observations are considered as the first migration. For years and regions with empty cells, too little data was available to determine the start of migration.

Supplementary Table S2. Start of plant phenological phases (Flowering or leaf unfolding) of apricot, common hazel, European larch, goat willow, horse-chestnut, silver birch and snowdrop in the regions warm, moderate and cool in the years 2000-2018 in Austria.

Supplementary Table S3. Robust regression models for predicting the start of amphibian migration of common frog and common toad. MAT = mean annual temperature [°C].

Supplementary Figure S1. Observations which mark the start of amphibian migration in the regions warm, moderate and cool in Austria. Regions are based on the mean annual air temperature (MAT) from 1971 to 2000 (zamg.ac.at). Map was created in ArcGIS 10.6.1 (esri.com/arcgis).

Supplementary Figure S2: Start of amphibian migration of common frogs and common toads between 2000-2018 - presented by regions warm (n=3), moderate (n=33) and cool (n=17).

Supplementary Table S1. Start of amphibian migration of common toads and common frogs in three climatic regions in the years 2000-2018. No data for common frogs in the warm region. Entries marked in bold indicate amphibians were sighted in several places that day, therefore two or more observations are considered as the first migration. For years and regions with empty cells, too little data was available to determine the start of migration.

Year	Warm region	Moderate region		Cool region	
	Comm. toad	Comm. toad	Comm. frog	Comm. toad	Comm. frog
2000		10 Mar	13 Mar	25 Mar	25 Mar
2001		08 Mar	08 Mar	11 Mar	15 Mar
2002		16 Mar	11 Mar		
2003		31 Mar	31 Mar	10 Apr	27 Mar
2004		**01 Apr**	31 Mar	07 Apr	02 Apr
2005		04 Mar	24 Mar	25 Mar	25 Mar
2006		**26 Mar**	24 Mar	10 Apr	05 Apr
2007		01 Mar	07 Mar	30 Mar	31 Mar
2008			27 Feb		
2009		24 Mar	27 Mar		
2010	19 Mar	21 Mar			26 Mar
2011	18 Mar	15 Mar	16 Mar		15 Mar
2012		12 Mar	19 Mar		**19 Mar**
2013		09 Apr			
2014		**17 Mar**			
2015		**23 Mar**			
2016	19 Mar	27 Mar			
2017			11 Mar		
2018		30 Mar			

Supplementary Table S2. Start of plant phenological phases (Flowering or leaf unfolding) of apricot, common hazel, European larch, goat willow, horse-chestnut, silver birch and snowdrop in the regions warm, moderate and cool in the years 2000-2018 in Austria.

Year	Apricot (Flowering)			Common hazel (Flowering)			European larch (Leaf unfolding)			Goat willow (Flowering)		
	Warm Region	Moderate Region	Cool Region	Warm Region	Moderate Region	Cool Region	Warm Region	Moderate Region	Cool Region	Warm Region	Moderate Region	Cool Region
2000		04 Mar	01 Apr		06 Feb	15 Feb		13 Mar	23 Mar		10 Feb	06 Mar
2001		17 Mar	13 Mar		17 Jan	12 Feb		16 Mar	29 Mar		20 Feb	07 Mar
2002		09 Mar			30. Jan			04 Mar			10 Feb	
2003		31 Mar	23 Mar		20 Feb	27 Feb		19 Mar	14 Apr		28 Feb	12 Mar
2004		31 Mar	28 Mar		17 Feb	23 Feb		01 Apr	10 Apr		04 Mar	10 Mar
2005		01 Apr	05 Apr		11 Mar	11 Mar		04 Apr	05 Apr		11 Mar	10 Mar
2006		03 Apr	09 Apr		17 Mar	18 Mar		28 Mar	15 Apr		09 Mar	19 Mar
2007		05 Mar	12 Mar		13 Jan	01 Feb		15 Mar	28 Mar		02 Feb	28 Feb
2008		24 Feb			22 Jan			19 Mar			12 Feb	
2009		31 Mar			15 Feb			01 Apr			02 Mar	
2010	25 Mar	22 Mar	19 Mar	24 Feb	27 Feb	03 Mar	01 Apr	31 Mar	06 Apr	20 Mar	14 Mar	28 Feb
2011	28 Feb	10 Mar	08 Mar	06 Feb	06 Feb	08 Feb	30 Mar	23 Mar	28 Mar	11 Mar	15 Feb	15 Feb
2012		18 Mar	18 Mar		26 Feb	01 Mar		26 Mar	17 Mar		01 Mar	08 Mar
2013		28 Mar			17 Feb			13 Apr			19 Mar	
2014		28 Feb			17 Jan			19 Mar			14 Feb	
2015		24 Mar			16 Feb			27 Mar			28 Feb	
2016	18 Mar	05 Mar		27 Jan	15 Jan		11 Mar	21 Mar		5 Mar	24 Feb	
2017		14 Mar			19 Feb			23 Mar			23 Feb	
2018		28 Mar			24 Jan			04 Apr			01 Mar	

Year	Horse-chestnut (Leaf unfolding)			Silver birch (Leaf unfolding)			Snowdrop (Flowering)		
	Warm Region	Moderate Region	Cool Region	Warm Region	Moderate Region	Cool Region	Warm Region	Moderate Region	Cool Region
2000		04 Apr	15 Apr		NA	NA		01 Feb	04 Feb
2001		29 Mar	17 Apr		NA	NA		29 Jan	08 Feb
2002		19 Mar			NA			02 Feb	
2003		21 Mar	14 Apr		03 Apr	17 Apr		08 Feb	28 Feb
2004		01 Apr	18 Apr		04 Apr	17 Apr		09 Feb	17 Feb
2005		02 Apr	03 Apr		04 Apr	19 Apr		04 Mar	15 Mar
2006		04 Apr	23 Apr		08 Apr	15 Apr		02 Mar	15 Mar
2007		02 Apr	08 Apr		21 Mar	30 Mar		23 Jan	18 Jan
2008		29 Mar			01 Apr			21 Jan	
2009		04 Apr			12 Mar			07 Feb	
2010	28 Mar	03 Apr	08 Apr	30 Mar	04 Apr	08 Apr	15 Feb	20 Feb	23 Feb
2011	28 Mar	21 Mar	02 Apr	02 Apr	27 Mar	21 Mar	18 Jan	28 Jan	06 Feb
2012		25 Mar	02 Apr		22 Mar	02 Apr		21 Feb	25 Feb
2013		16 Apr			15 Apr			02 Feb	
2014		23 Mar			14 Mar			18 Jan	
2015		09 Apr			09 Apr			17 Jan	
2016	30 Mar	02 Apr		31 Mar	04 Apr		29 Jan	15 Jan	
2017		27 Mar			25 Mar			16 Feb	
2018		09 Apr			07 Apr			28 Jan	

Supplementary Table S3. Robust regression models for predicting the start of amphibian migration of common frog and common toad. MAT = mean annual temperature [°C].

Number	Predictor variables	Calculation	RMSE	Adj. R^2	R^2
**Common frog**					
	Empty (nullmodel)	y ~ 1	9.95	-	-
Models where MAT (°C) not addressed yellow, models for specific sites where MAT (°C) is known marked blue					
	Common hazel	y = 61.65 + 0.37 * C_h	7.67	0.4434	0.4687
		y = 97.12 + 0.30 * C_h – 4.5 * MAT	7.00	0.5659	0.6053
1	Goat willow	y = 44.31 + 0.61 * G_w	6.47	0.5511	0.5715
		y = 70.6 + 0.52 * G_w – 2.94 * MAT	6.26	0.5972	0.6338
	Comm. hazel, goat willow	y = 45.29 + 0.56 * G_w + 0.03 * C_h	7.01	0.53	0.57
		y = 73.39 + 0.43 * G_w + 0.06 * C_h – 3.08 * MAT	6.76	0.58	0.64
2	Apricot	y = 24.06 + 0.69 * A	4.95	0.7415	0.7538
		y = 52.65 + 0.64 * A – 3.27 * MAT	4.22	0.8365	0.8529
	Apricot, common hazel	y = 29.18 + 0.58 * A + 0.08 * C_h	6.27	0.6772	0.7065
		y = 56.12 + 0.52 * A + 0.06 * C_h – 2.93 * MAT	6.05	0.7206	0.7587
Models derived therefrom for moderate (5°C ≤ MAT < 7°C) and cold region (7°C ≤ MAT < 9°C)					
1a, 1b	Goat willow	Moderate: 47.08 + 0.52 * G_w; cold: 52.96 + 0.52 * G_w			
2a, 2b	Apricot	Moderate: 26.49 + 0.64 * A; cold: 33.03 + 0.64 *A			
**Common toad**					
	Empty (nullmodel)	y ~ 1	11.25	-	-
Models (MAT (°C) not addressed)					
	Apricot	y = 44.84 + 0.46 * A	10.1	0.2212	0.2512
3	Goat willow	y = 43.38 + 0.65 * G_w	8.11	0.47	0.49
	Apricot, Goat willow	y = 39.91 + 0.37 * G_w + 0.24 *A	9.87	0.32	0.38

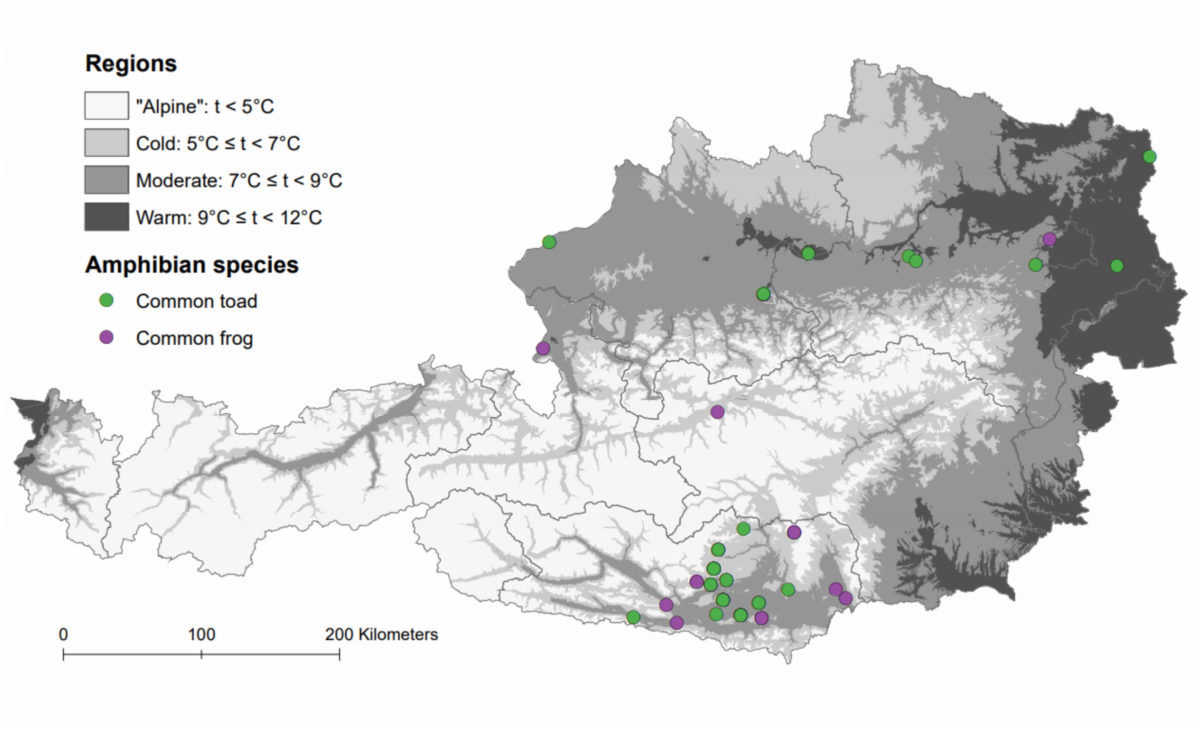

Supplementary Figure S1. Observations which mark the start of amphibian migration in the regions warm, moderate and cool in Austria. Regions are based on the mean annual air temperature (MAT) from 1971 to 2000 (zamg.ac.at). Map was created in ArcGIS 10.6.1 (esri.com/arcgis).

.

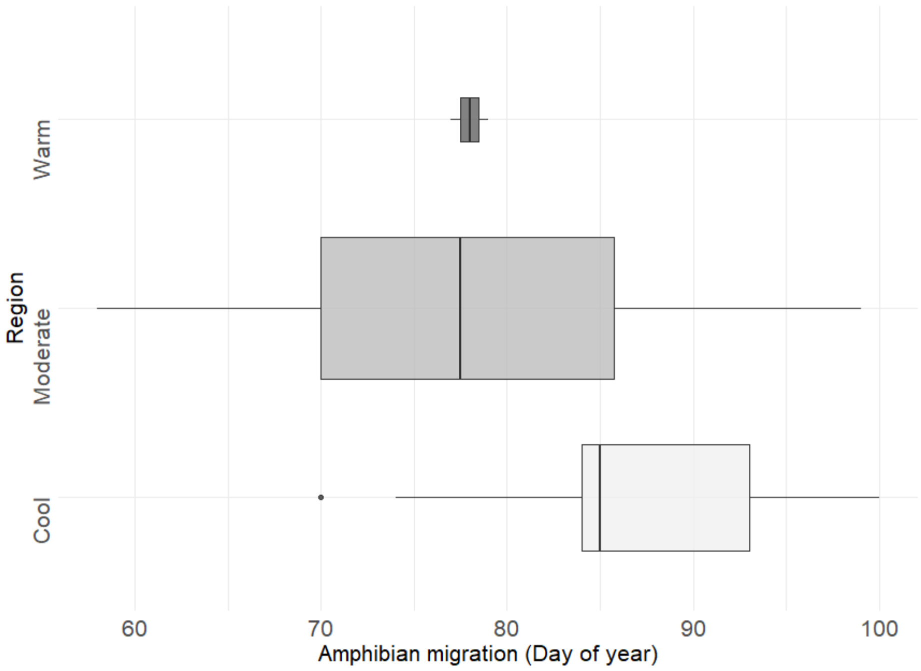

Supplementary Figure S2: Start of amphibian migration of common frogs and common toads between 2000-2018 - presented by regions warm (n=3), moderate (n=33) and cool (n=17).

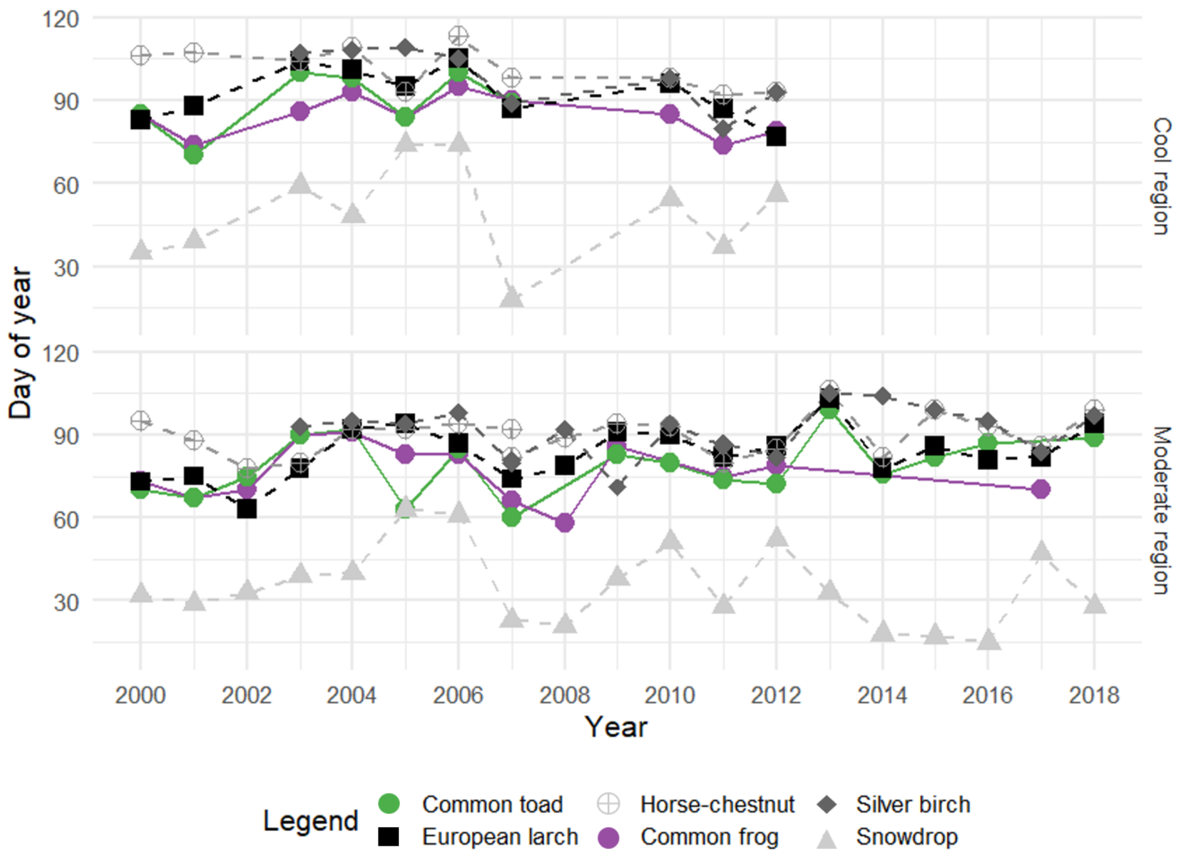

Supplementary Figure S3: Phenological time series of amphibian species common frog and common toad and plant species European larch, horse-chestnut, silver birch and snowdrop.
